# Book review: “Glial physiology and pathophysiology” by Alexei Verkhratsky and Arthur Butt, Wiley-Blackwell, 2013

**DOI:** 10.3389/fnsys.2014.00017

**Published:** 2014-02-11

**Authors:** Leif Hertz

**Affiliations:** Department of Clinical Pharmacology, College of Basic Medical Sciences, China Medical UniversityShenyang, China

**Keywords:** astrocytes, glial diseases, microglia, NG2 cells, oligodendrocytes, peripheral glia

The textbook “Glial Physiology and Pathophysiology” by Alexei Verkhratsky and Arthur Butt is aimed at researchers and clinicians as a detailed and comprehensive source of readily obtainable information. It addresses readers wanting to get a systematic, brief and correct overview of glial function in healthy and diseased brain. That ought to be each and every neuroscientist and neurologist. In 2014 it is no longer okay for any neurologist or neuroscientist to be unaware of basic features of glial functions and dysfunction. Occasionally it is difficult to obtain correct information about glial functions because of less than perfect means to study these functions. Thus, young rat/mouse brain (the first 3–4 weeks) is completely different from its adult counterpart, with un- or under-developed glial-neuronal interactions, but often used for glial studies on account of greater simplicity. Another problem is that morphological (immunohistochemical or *in situ* hybridization) techniques unpredictably quite often fail to reveal glial localization, partly due to the fact that the generally used glial fibrillary protein (GFAP) is not an ideal marker for astrocytes, and the book makes references to better ones. It also discusses important new techniques based on insertion of fluorescent compounds cell-specifically in the genome, which now makes it possible to obtain freshly isolated neurons, oligodendrocytes, and microglia directly from transgenic animals. These cells are ideal for studies of gene expression and drug-induced changes in the expression, but probably not yet quite good enough for more functional studies. A third difficulty is the often uncritical use of glial cell cultures. The two latter points are discussed in detail in a recent paper by Peng et al. (2013). This paper shows that in the case of astrocytes, primary cultures *can* be prepared in such a way that they provide correct information, as indicated by 100% identical results in cultures used by this group and freshly isolated cells, when comparisons were made. A fourth probable reason for error is failure by some researchers to pay attention to the finding by Kostyuk (1984) that Ca^2+^ channel function requires metabolic energy. The present authors are well aware of these problems in glial research, most of which are discussed, and they have been remarkably successful in steering away from data tainted by these sources of errors. Accordingly, the errors are few, the only significant one being an undererstimate of the astrocytic fraction of total glucose oxidation in brain cortex (should be 20–25%).

The authors emphasize the dual function of the central nervous system as consisting of *neurons* interacting one to one, and *astrocytes* acting as a functional syncytium (endorsing views of *both* Golgi and Ramon y Cajal). The combined function of the two closely collaborating systems is essential for such functions as memory and diseases affecting memory, which include glial abnormalities (Gibbs et al., 2009). Two specific systems are critically dependent upon interactions between astrocytes and neurons and are discussed in some detail: The glutamatergic/GABAergic system utilizes exclusively glutamate formed from glucose by astrocytes, conversion of glutamate to glutamine, which has no transmitter activity, transfer of glutamine from astrocytes to neurons in the glutamine glutamate(GABA)-glutamine cycle, and exocytotic release from neurons. The major fractions of the released transmitters return to astrocytes, from where most, but not all, is converted to glutamine and re-returned to neurons, whereas ~20% is oxidized in astrocytes, requiring de novo synthesis from glucose. This system operates in brain cortex at a rate similar to that of glucose metabolism. A second system discussed in detail, but only complete if including a 2012 paper (Bay and Butt, 2012) by Butt (the second author), studying neuronal-astrocytic interactions clearing excess extracellular K^+^, released to the extracellular space during propagation of the action potential. This K^+^ is first accumulated into astrocytes by the powerful, K^+^-stimulated astrocytic Na^+^, K^+^-ATPase, and then released again via Kir1.4 (probably after redistribution in the astrocytic syncytium) and re-accumulated by the neuronal Na^+^, K^+^-ATPase, which is not stimulated by excess K^+^. What was not known when the textbook was published is the possible essential contribution of K^+^-stimulated glycogenolysis for the astrocytic uptake (Xu et al., 2013). Glycogen, a high-energy compound is extremely well suited for homeostasis (a major astrocytic task), because it is stable at control concentrations of K^+^ concentration but broken down at even the slightest extracellular K^+^ increase (Hof et al., 1988). Finally the essential astrocytic contributions to synaptogenesis are discussed in detail. In the adult nervous system transmitter effects on astrocytic [Ca^2+^]_i_ are of great importance, whereas astrocytic transmitter release, with the exception of ATP, probably has been overestimated previously.

*Oligodendrocytes* and *NG2 cells* are also thoroughly described. The main role of oligodendrocytes is production of myelin, which greatly enhances the rate of nerve conduction, as well as a contribution to formation and maintenance of the nodes of Ranvier, which are essential for rapid saltatory conduction and thus for normal cognitive function. Figure 5.6 in the book provides an excellent illustration of spatial relationship between myelin, axon and astrocytes, but provides no information how K^+^ exiting the axon in the juxtanodal region reaches the astrocyte, which serves to remove it. However, under demyelinating diseases it is mentioned that all neural cell types are involved in the disease. Most channels, transporters, etc. resemble those in astrocytes. Glutamate receptor activation is important during development but places oligodendrocytes at risk for excitotocity. NG-2 cells are oligodendrocyte precursors, but probably also have other roles. This may also apply to perineuronal oligodendrocytes.

*Microglia* are discussed as mesodermal/mesenchymal cells entering the CNS during development. They patrol and supervise brain parenchyma and rapidly respond to injury and atrophic changes. But they also help pruning during development, and their aging facilitates neurodegeneration.

*Peripheral glia is* understudied but known to show prominent Ca^2+^ signaling, ion channels and neurotransmitter receptors. Enteric glia plays a major role in intestinal diseases. Many other diseases with huge glial involvement are briefly discussed, which should wet the readers' appetite for further information in cited and non-cited specialized articles. Both the detailed description of astrocyte and oligodendrocyte function and the cursory treatment of diseases with heavy glial involvement make this book indispensable.
